# Supplementary material for: Comparative transcriptome analysis uncovers different heat stress responses in heat-resistant and heat-sensitive jujube cultivars
Source: PLoS One. 2020 Sep 21;15(9):e0235763. doi: 10.1371/journal.pone.0235763 (PMC7505471; doi:10.1371/journal.pone.0235763)
Supplement: Attachment — Submitted filename: PONE-D-20-01047_reviewer marked.pdf [file pone.0235763.s008.pdf]

PLOS ONE

Comparative Transcriptome Analysis Uncovers Different Heat Stress Responses in Heat-resistant and Heat-sensitive Jujube Cultivars

--Manuscript Draft--

**Manuscript Number:**	PONE-D-20-01047
**Article Type:**	Research Article
**Full Title:**	Comparative Transcriptome Analysis Uncovers Different Heat Stress Responses in Heat-resistant and Heat-sensitive Jujube Cultivars
**Short Title:**	Comparative Transcriptome Analysis Uncovers Different Heat Stress Responses in Two Jujube Cultivars
**Corresponding Author:**	jin juan<br>Xinjiang Academy of Agricultural Sciences<br>Urumqi City, CHINA
**Keywords:**	jujube; heat stress; RNA-seq; heat shock proteins; transcriptional factors; ubiquitin-protein ligase genes
**Abstract:**	Jujube (Ziziphus jujuba Mill.) is an economically and agriculturally significant fruit crop and is widely cultivated throughout the world. Heat stress has recently become a primary abiotic stressor limiting the productivity and growth of jujube, as well as other crops. There are few studies, however, that have performed transcriptome profiling of jujube when it is exposed to heat stress. In this study, we observed the physiochemical changes in heat-resistant jujube cultivar ‘HR’ and the heat-sensitive cultivar ‘HS’ and twenty-four cDNA libraries generated from ‘HR’ and ‘HS’ were built with a transcriptome assay. A total of 1880, 4350 and 5435 differentially expressed genes were identified in ‘HR’ with 2390, 2998, and 4064 genes detected in ‘HS’ after heat stress for 1, 3 and 5d, respectively. The expression pattern of nine genes was validated by qRT-PCR. After gene ontology and KEGG enrichment analysis, the DEGs encoding heat shock proteins, transcriptional factors, and ubiquitin-protein ligase genes were found to be closely involved in heat stress response. These results will provide useful information for elucidating the molecular mechanism underlying heat stress in different jujube cultivars.
**Order of Authors:**	juan jin
	lei yang
	yu ding fan
	xin xu liu
	qing hao
**Additional Information:**	
**Question**	**Response**
**Financial Disclosure**<br><br>Enter a financial disclosure statement that describes the sources of funding for the work included in this submission. Review the submission guidelines for detailed requirements. View published research articles from *PLOS ONE* for specific examples.<br><br>This statement is required for submission and **will appear in the published article** if the submission is accepted. Please make	This research was supported financially by the National Natural Science Foundation of China (Grant # 31801815).

sure it is accurate.<br><br>**Unfunded studies**<br>Enter: *The author(s) received no specific funding for this work.*<br><br>**Funded studies**<br>Enter a statement with the following details:<br>• Initials of the authors who received each award<br>• Grant numbers awarded to each author<br>• The full name of each funder<br>• URL of each funder website<br>• Did the sponsors or funders play any role in the study design, data collection and analysis, decision to publish, or preparation of the manuscript?<br>• **NO** - Include this sentence at the end of your statement: *The funders had no role in study design, data collection and analysis, decision to publish, or preparation of the manuscript.*<br>• **YES** - Specify the role(s) played.<br><br>* typeset	
**Competing Interests**<br><br>Use the instructions below to enter a competing interest statement for this submission. On behalf of all authors, disclose any competing interests that could be perceived to bias this work—acknowledging all financial support and any other relevant financial or non-financial competing interests.<br><br>This statement **will appear in the published article** if the submission is accepted. Please make sure it is accurate. View published research articles from *PLOS ONE* for specific examples.	The authors have declare that they have no competing interests.

Powered by Editorial Manager® and ProduXion Manager® from Aries Systems Corporation

**NO authors have competing interests**<br><br>Enter: *The authors have declared that no competing interests exist.*<br><br>**Authors with competing interests**<br><br>Enter competing interest details beginning with this statement:<br><br>*I have read the journal's policy and the authors of this manuscript have the following competing interests: [insert competing interests here]*<br><br>* typeset	
**Ethics Statement**<br><br>Enter an ethics statement for this submission. This statement is required if the study involved:<br><br>• Human participants<br>• Human specimens or tissue<br>• Vertebrate animals or cephalopods<br>• Vertebrate embryos or tissues<br>• Field research<br><br>Write "N/A" if the submission does not require an ethics statement.<br><br>General guidance is provided below. Consult the submission guidelines for detailed instructions. **Make sure that all information entered here is included in the Methods section of the manuscript.**	N/A

Powered by Editorial Manager® and ProduXion Manager® from Aries Systems Corporation

**Format for specific study types**<br><br>**Human Subject Research (involving human participants and/or tissue)**<br>• Give the name of the institutional review board or ethics committee that approved the study<br>• Include the approval number and/or a statement indicating approval of this research<br>• Indicate the form of consent obtained (written/oral) or the reason that consent was not obtained (e.g. the data were analyzed anonymously)<br><br>**Animal Research (involving vertebrate animals, embryos or tissues)**<br>• Provide the name of the Institutional Animal Care and Use Committee (IACUC) or other relevant ethics board that reviewed the study protocol, and indicate whether they approved this research or granted a formal waiver of ethical approval<br>• Include an approval number if one was obtained<br>• If the study involved *non-human primates*, add *additional details* about animal welfare and steps taken to ameliorate suffering<br>• If anesthesia, euthanasia, or any kind of animal sacrifice is part of the study, include briefly which substances and/or methods were applied<br><br>**Field Research**<br><br>Include the following details if this study involves the collection of plant, animal, or other materials from a natural setting:<br>• Field permit number<br>• Name of the institution or relevant body that granted permission	
**Data Availability**<br><br>Authors are required to make all data underlying the findings described fully available, without restriction, and from the time of publication. PLOS allows rare exceptions to address legal and ethical concerns. See the PLOS Data Policy and FAQ for detailed information.	Yes - all data are fully available without restriction

A Data Availability Statement describing where the data can be found is required at submission. Your answers to this question constitute the Data Availability Statement and **will be published in the article**, if accepted.<br><br>**Important:** Stating 'data available on request from the author' is not sufficient. If your data are only available upon request, select 'No' for the first question and explain your exceptional situation in the text box.<br><br>Do the authors confirm that all data underlying the findings described in their manuscript are fully available without restriction?	
**Describe where the data may be found in full sentences. If you are copying our sample text, replace any instances of XXX with the appropriate details.**<br><br>• If the data are **held or will be held in a public repository**, include URLs, accession numbers or DOIs. If this information will only be available after acceptance, indicate this by ticking the box below. For example: *All XXX files are available from the XXX database (accession number(s) XXX, XXX.*).<br>• If the data are all contained **within the manuscript and/or Supporting Information files**, enter the following: *All relevant data are within the manuscript and its Supporting Information files.*<br>• If neither of these applies but you are able to provide **details of access elsewhere**, with or without limitations, please do so. For example:<br><br>*Data cannot be shared publicly because of [XXX]. Data are available from the XXX Institutional Data Access / Ethics Committee (contact via XXX) for researchers who meet the criteria for access to confidential data.*<br><br>*The data underlying the results presented in the study are available from (include the name of the third party*	The RNA-seq data were deposited at the NCBI Gene Expression Omnibus (GEO) database under accession numbers GSE136383 and GSE136047.

and contact information or URL).<br>• This text is appropriate if the data are owned by a third party and authors do not have permission to share the data.<br><br>* typeset	
Additional data availability information:	Tick here if the URLs/accession numbers/DOIs will be available only after acceptance of the manuscript for publication so that we can ensure their inclusion before publication.

 Click here to access/download;Manuscript;Manuscript.docx

Comparative Transcriptome Analysis Uncovers Different Heat Stress Responses in Heat-resistant and Heat-sensitive Jujube Cultivars

Juan Jin[1], Lei Yang[1], Dingyu Fan[1], Xuxin Liu[2] and Qing Hao[1*]

[1] Institute of Horticultural crops, Xinjiang Academy of Agricultural Sciences, Urumqi 830091, China;
[2] Xinjiang Agricultural Vocational Technical College, Changji 831100, China

Juan Jin and Lei Yang contributed equally to this work.

*haoqingxj@sohu.com

Abstract: Jujube (*Ziziphus jujuba* Mill.) is an economically and agriculturally significant fruit crop and is widely cultivated throughout the world. Heat stress has recently become a primary abiotic stressor limiting the productivity and growth of jujube, as well as other crops. There are few studies, however, that have performed transcriptome profiling of jujube when it is exposed to heat stress. In this study, we observed the physiochemical changes in heat-resistant jujube cultivar ‘HR’ and the heat-sensitive cultivar ‘HS’ and twenty-four cDNA libraries generated from ‘HR’ and ‘HS’ were built with a transcriptome assay. A total of 1880, 4350 and 5435 differentially expressed genes were identified in ‘HR’ with 2390, 2998, and 4064 genes detected in ‘HS’ after heat stress for 1, 3 and 5d, respectively. The expression pattern of nine genes was validated by qRT-PCR. After gene ontology and KEGG enrichment analysis, the DEGs encoding heat shock proteins, transcriptional factors, and ubiquitin-protein ligase genes were found to be closely involved in heat stress response. These results will provide useful information for elucidating the molecular mechanism underlying heat stress in different jujube cultivars.

Introduction

Plants have to deal with a broad array of environmental conditions, such as drought, salt, low temperatures, high temperatures, nutrition deficiency, and heavy metals, which negatively affect the growth and development of plants[1,2]. These abiotic stressors induce a number of different responses in plants, including biochemical, physiological, molecular, anatomical, and morphological changes[3,4]. Because of the increasing temperatures worldwide, heat stress has become one of the primary environmental factors restricting the distribution and cultivation of crops globally, thus leading to significant reductions in yield, growth, and mortality[5]. Hence, it is an effective way to seek for the suitable heat-resistant cultivar in nature and explore its mechanism of plant stress resistance.

Plants have evolved multiple strategies to combat high temperatures in an effort to reduce the negative effects of heat stress on crop growth and development [6]. At the cellular, physiological, and cellular levels, the generation of phytohormones, antioxidants and osmoprotectants, as well as dynamic membrane regulation, and stomatal closure are associated with plant adaptive responses against heat stress [7,8]. Heat stress changes the way genes involved in signaling pathways, as well as transcriptional control and the expression heat shock proteins at the molecular level[9]. New research has found that levels of gene expression induced by heat stress are regulated by transcriptional networks as well as the transcription factors (TFs) in plants after translational regulation[10]. For example, heat shock proteins (HSPs) are known as target genes for TFs responding to heat stress[11,12]. Many HSPs function as molecular chaperones[13] and serve important roles in the regulation of the protein quality by renaturing various proteins that have been denatured by heat stress[14]. In peanuts, accumulation of small HSPs could improve its resistance to heat[15].

In recent years, transcriptome sequencing has become an important technique for identifying stress-related genes and finding the multiple biological pathways[16]. It has been successfully used to reveal mechanisms related to abiotic stress responses in many species, such as Chinese kale (*Brassica alboglabra*)[17], *Pyropia haitanensis*[18], Chinese cabbage (*Brassica rapa* ssp. Chinensis)[19], sweet maize (*Zea mays* L.)[20], spinach (*Spinacia oleracea* L.)[21], pepper (*Capsicum annum* L.)[22]. Jujube

(*Ziziphus jujuba* Mill.) is a traditional fruit crop, native to China, which belongs to the Rhamnaceae family and widely grown in Asian tropical and subtropical areas[23]. Approximately 3.25 million hectares are cultivated in China [24]. High temperature caused serious loss of quality and production during the jujube growth especially in the open field cultivation. However, little knowledge about the molecular studies on jujube under heat stress is uncovered.

In this study, we obtained two differently heat-resistant jujube cultivars and carried out RNA-sequencing analysis to explore the transcriptional changes between the two jujube cultivars under heat stress. Our objective was to identify differentially expressed genes involved in heat stress on jujube, and potential heat stress-related genes including heat shock proteins, transcriptional factors, and ubiquitin-protein ligase genes were detected. This study provides a theoretical basis for elucidating the molecular mechanisms on heat tolerance in jujube.

Materials and Methods

Plant materials, heat Stress treatments and sample collection

Green stem cuttings of 'HR' and 'HS' (*Ziziphus jujuba* Mill.) were rooted in pots with a mixture of 1 perlite: 1 vermiculite: 1 river sand (v/v/v) and grown in a greenhouse under mist conditions. When the cuttings were rooted, they were repotted into larger pots, and grown in a greenhouse at 70-80% relative humidity. The temperature was almost kept at 30°C and the maximum light intensity was about 800 μmol $m^{-2}s^{-1}$ during the daytime, the temperature was maintained at 20°C during the night. About 6 weeks later, young seedlings of 'HR' and 'HS' were transferred to a controlled environment room (14h/10h light/dark, 30/20°C day/night cycle, 70-80% relative humidity, and light intensity at 600 μmol $m^{-2}s^{-1}$). Seedlings with eight true leaves of 'HR' and 'HS' were then treated at 45°C in the same controlled environment room (except for temperature, the other conditions were the same as above) from 8:00 am to 22:00 pm. After 0 (control), 1d, 3d, 5d of heat treatment, the 6th true leaves (from the top or bottom) were then collected from all three different plants in each treatment and control at 16:00 pm. Samples were immersed in liquid nitrogen and stored at -80°C for subsequent experimentation.

Physiological measurements and ultra-structural observation

Before the transcriptome study, we measured three physiological indexes, including electrolyte leakage, malondialdehyde (MDA), and proline in order to analyze the physiological changes of two jujube cultivars subjected to heat stress. Relative electrolyte leakage (relative electrolyte conductivity; REC) was used to assess the cell membrane permeability as described by Yang et al.[25]. Lipid peroxidation was determined by measuring the amount of malondialdehyde (MDA) content according to the methods of Heath[26]. Proline content was determined using the sulfosalicylicaid method[27].

For ultra-structural observation, the leaf tissue adjacent to the vein were cut into 1 mm × 2 mm and fixed immediately by immersion in 4 % glutaraldehyde overnight, then washed with 0.1 M PBS (pH7.4) for 15 min three times. After post fixation in 1 % osmium tetroxide for 5 h at 20 °C, the fragments were washed with 0.1 M PBS for 15 min three times and gradually dehydrated in ethanol (30, 50, 70, 80, 90, 95 and 100 % ethanol for 1 h), then immersed in 3 ethanol: 1acetone (v/v), 1 ethanol: 1acetone (v/v), 1 ethanol: 3 acetone (v/v) for 30 min, respectively. The fixed samples were embedded in SPIpon812 epoxy matrix (SPI, Chem) and observed by transmission electron microscopy (HT7700, Hitachi, Tokyo, Japan).

RNA extraction, cDNA library construction and Illumina sequencing

Total RNA was extracted from jujube leaf samples using the RNAprep Pure Plant Kit (Tiangen, Beijing, China) following the manufacturer's instructions. The purity of the extracted RNA was assessed by NanoDrop 1000 spectrophotometer (Thermo Fisher Scientific, Wilmington, DE, USA). RNA concentration was performed by using Qubit® 2.0 Flurometer (Life Technologies, Carlsbad, CA, USA).

RNA integrity was checked by the RNA Nano 6000 Assay Kit of the Agilent Bioanalyzer 2100 system (Agilent Technologies, Santa Clara, CA, USA).

RNA sequencing libraries were performed at Biomarker Technologies Corporation (Beijing, China) following instructions similar to Guo et al. [17]. Briefly, after enrichment and purification with oligo (dT)-rich magnetic beads, mRNA was cleaved into short fragments. Then first- and second-strand cDNA were synthesized using the mRNA fragments as templates. The cDNA was purified by AMPure XP beads and resolved with elution buffer for end reparation and single nucleotide adenine addition, and the short fragments were connected with adapters. Then the suitable fragments were selected as templates for PCR amplification. Finally, the twenty-four cDNA libraries were sequenced using an Illumina HiSeq™ 2500. The RNA-seq data were deposited at the NCBI Gene Expression Omnibus (GEO) database under accession numbers GSE136383 and GSE136047.

RNA-seq reads mapping and transcript assembly

After trimming adapter sequences, removing low quality reads and unknown nucleotides larger than 5%, clean reads were filtered from the raw reads. Cleaned RNA-seq reads were then mapped to the jujube reference genome retrieved from the NCBI Database (http://www.ncbi.nlm.nih.gov/genome/)[28]using Bowtie 2 (http://bowtie-bio.sourceforge.net/index.shtml) [29] and TopHat 2 (http://ccb.jhu.edu/software/tophat/index.shtml)[30]. Then the Sequence Alignment Map (http://samtools.sourceforge.net)[31] files were generated by TopHat2 and subsequently transcripts were assembled by Cufflinks (http://cufflinks.cbcb.umd.edu/)[32].

Differentially expressed genes and functional analysis

Differentially expressed genes (DEGs) of different libraries were analyzed using the FPKM (the fragments per kilobase of exon per million fragments mapped reads) method and the Bioconductor software package edgeR was used to screen out the DEGs[33]. Genes were determined to be differentially expressed based on the fold change ($FC \geq 2$ or ≤ 0.5) and a false discovery rate ($FDR \leq 0.01$).

Gene ontology (GO) and Kyoto Encyclopedia of Gene and Genomes (KEGG) enrichment analysis of the DEGs were performed using the GOseq R packages[34] and KOBAS software[35], respectively, and the KEGG terms with corrected $P<0.05$ were considered as significantly enriched.

Validation of RNA-seq by quantitative real-time PCR (qRT-PCR)

The qRT-PCR was performed on RNA extracted from leaf samples of both cultivars at the four heat treatment time points as described by Bu et al.[36] using the *ZjActin* as the internal control to normalize the expression data. Primers used for qRT-PCR were listed in **S1 Table**. We used the same samples of RNA for both the qRT-PCR verification and the RNA-seq. 1 μg of RNA was reversely transcribed using OneScript® Two-Step RT-PCR Kit (Applied Biological Materials (ABM), Vancouver, Canada) according to the instructions of the manufacturer. We performed the experiment on the LightCycler96 with BrightGreen Express 2X qPCR MasterMix (ABM, Canada), according to the following PCR program: 2 min of denaturing at 95°C, 5 s of denaturing at 95°C, performed 40 times, 30 s of extension and annealing at 60°C. Gene expression was calculated by the $2^{-\Delta\Delta Ct}$ method[37]. All qRT-PCR were repeated with three technical and three biological replicates.

Statistical analysis

One-way analysis of variance (ANOVA) was conducted using SPSS 17.0 statistical software (SPSS, IL, USA). We used Duncan's Multiple Range Test to analyze the differences among means, and considered results to be significant at a p-value ≤ 0.05.

Results and Discussion

Physiochemical changes of two jujube cultivars in response to heat stress

To describe the heat tolerance phenotype of the heat-resistant 'HR' and heat-sensitive 'HS' jujube cultivars, young jujube seedlings with uniform growth during the eight-leaf stage were exposed to 45 °C of heat stress for 0, 1, 3, and 5 d, respectively. Our ultrastructure observation indicated that (a) the nucleoli of heat-resistant jujube cultivar 'HR' and heat-sensitive jujube cultivar 'HS' were clearly visible, chloroplasts were long spindles that regularly grew near the cell wall and contained white starch grains in the control treatment (0 d); (b) the nucleoli of two jujube cultivars gradually disappeared as the heat stress time extended, but the nuclear membranes remained intact; (c) chloroplast of 'HR' could basically maintain a relatively normal shape, and the starch grains were faintly visible as the heat stress time increased; however, the chloroplast of 'HS' started to swell and became round, detached from the cell wall and floated into the cell, and the starch granules gradually disappeared; (d) osmiophilic granules of 'HS' were significantly more than that of 'HR' after heat stress at 3, 5d (**Figure 1A**).

In order to further confirm the difference in heat tolerance between the heat-resistant jujube cultivar 'HR' and the heat-sensitive jujube cultivar 'HS', we measured the electrolyte leakage, MDA and proline content. Compared with the control treatment (0 d), there was no significant difference in the relative electrolyte leakage and MDA content in 'HR' leaves after 1, 3 d of heat stress, while exposure to heat stress for 5 days significantly increased the relative electrolyte leakage and MDA content by 18.04% and 36.14%, respectively (**Figure 1B,C**). For heat-sensitive jujube cultivar 'HS', exposure to heat stress for 1, 3 days significantly increased the relative electrolyte leakage by 25.36%, 37.97%, respectively, and the MDA content were significantly increased by 34.93%, 75.69% and 117.29% after 1, 3,5 d of heat stress, respectively.

Compared with the control treatment (0 d), exposure to heat stress for 3, 5 days significantly increased the proline content in the leaves of 'HR' and 'HS', among which the proline content of 'HR' were increased by 59.53%, 211.75%, respectively, and the proline content of 'HS' were increased by 54.83% ,165.09%, respectively. No significant differences were observed in the proline content of the two jujube cultivars between the 1d of heat stress and the control treatments (**Figure 1D**). In addition, the proline content of 'HR' was always higher than that of 'HS' among all the experimental treatments. These results indicated that 'HS' was sensitive while 'HR' was tolerant to heat stress.

Illumina Sequencing and Alignment to the Reference Genome

In order to determine the transcriptome profiles of 'HR' and 'HS' in response to heat stress, we performed RNA-Seq analyses on 'HR' and 'HS' at 0, 1, 3, and 5 d. Three biological replicates were made at each time point for both cultivars. We established 24 RNA cDNA libraries (HR-0-a, HR-0-b, HR-0-c; HR-1-a, HR-1-b, HR-1-c; HR-3-a, HR-3-b, HR-3-c; HR-5-a, HR-5-b, HR-5-c; HS-0-a, HS-0-b, HS-0-c; HS-1-a, HS-1-b, HS-1-c; HS-3-a, HS-3-b, HS-3-c; HS-5-a, HS-5-b, HS-5-c). After filtering and trimming, 22,595,993-32,752,424 clean reads (the percentage of Q30 and GC being 91.25–94.79% and 44–45.97%) were obtained (**S2 Table**), Most reads (81.91–87.76%) could be mapped to the jujube reference genome, among which 71.08–76.71% were uniquely mapped ones. Box-plots displayed the range of the fragments per kilobase of exon per million fragments mapped (FPKM) values in all the 24 libraries (**Figure 2A**). Sample clustering analysis presented 4 Clusters. We found that Cluster 1 only gathered samples of 'HR', Cluster 2 assembled mostly samples of 'HS', Cluster 3 and 4 grouped about the same number of samples in both 'HR' and 'HS', these results indicated that the gene expression profile in 'HR' is quite different from that in 'HS' after 5 d of heat stress (**Figure 2B**).

Explorations of differentially expressed genes between 'HR' and 'HS' cultivars

To identify the number of DEGs between 'HR' and 'HS' after 0, 1, 3, 5d of heat stress, we analyzed the gene expression patterns using the DEGseq[33] based on the Fold Change ≥2 and FDR <0.01.

As shown in Table 1, a total of 1880, 4350 and 5435 annotated genes were identified in ‘HR’ after heat stress for 1, 3 and 5d, respectively; among which, 1078 up-regulated genes and 802 down-regulated genes were identified in the HR0 vs HR1 comparison; in the HR0 vs HR3 comparison, the number of up-regulated genes and down-regulated genes was 2077 and 2273, respectively; in the HR0 vs HR5 comparison, the numbers were 2315 and 3120, respectively (**S3 Table**).Meanwhile, There were 2390, 2998, and 4064 annotated genes differentially expressed in ‘HS’ after heat stress for 1, 3 and 5d, respectively; in the HS0 vs HS1 comparison, the number of up-regulated genes and down-regulated genes was 1035 and 1355, respectively; in the HS0 vs HS3 comparison, 1327 genes were up-regulated and 1671 genes were down-regulated; in the HS0 vs HS5 comparison, there were 1759 up-regulated genes and 2305 down-regulated genes.

Venn diagram analysis showed that 487 up-regulated and 547 down-regulated genes overlapped in the HR0 vs HR1, HR0 vs HR3, HR0 vs HR5 comparisons, whereas 555 common genes were found to be up-regulated and 951 down-regulated in the HS0 vs HS1, HS0 vs HS3, HS0 vs HS5 comparisons (**Figure 3**).

Furthermore, we identified the genes expressed under the same heat stress time period between ‘HR’ and ‘HS’ (Table 1). In the control treatment (0 d), 179 genes were up-regulated and 158 genes were down-regulated in the HR0 vs HS0 comparison; After being subjected to heat stress, 203 genes were up-regulated and 402 genes were down-regulated in the HR1 vs HS1 comparison; 388 genes were up-regulated and 444 genes were down-regulated in the HR3 vs HS3 comparison; and 860 genes were up-regulated and 543 genes were down-regulated in the HR5 vs HS5 comparison. Venn diagram results showed that only 5 genes were down-regulated in the four comparisons (**Figure 4**).

Table 1. The number of DEGs in different comparisons

DEGs	All DEGs		Up-regulated	Down-regulated
	Annotated	New discovered		
HR0d_vs_HR1d	1880	56	1078	802
HR0d_vs_HR3d	4350	95	2077	2273
HR0d_vs_HR5d	5435	102	2315	3120
HS0d_vs_HS1d	2390	68	1035	1355
HS0d_vs_HS3d	2998	85	1327	1671
HS0d_vs_HS5d	4064	86	1759	2305
HR0d_vs_HS0d	337	18	179	158
HR1d_vs_HS1d	605	23	203	402
HR3d_vs_HS3d	832	39	388	444
HR5d_vs_HS5d	1403	37	860	543

Functional Analysis of DEGs between ‘HR’ and ‘HS’ Cultivars

To functionally annotate the DEGs between the two cultivars after different time of heat stress, we aligned the DEGs against the Gene Ontology (GO) and Kyoto and Encyclopedia of Genes and Genomes (KEGG) database. For the GO analysis, the DEGs were summarized to three categories: Biological Process (BP), Cell Component (CC) and Molecular Function (MF). **Figure 5** shows the GO classification results of the DEGs, 3934 DEGs from different stress time points in ‘HR’ and 2965 DEGs from different stress time points in ‘HS’ could be categorized into 52 functional groups (**Figure 5 A, B, S4 Table**). Among these categories, within the biological process, “metabolic process” (2282), “cellular process” (1884), “single-organism process” (1761), “response to stimulus” (817) and “biological regulation” (737) were significantly enriched, within the molecular function, “catalytic activity” (2261) and “binding” (1769) were significantly enriched at all heat stress time points in ‘HR’ (**Figure 5A**). In ‘HS’, “metabolic process” (1747), “cellular process” (1435), “single-organism process” (1336), “response to stimulus” (657) and “biological regulation” (534) were also significantly enriched in the biological process, within the molecular function, “catalytic activity” (1736) and “binding” (1307) were significantly enriched at all heat stress time points in ‘HS’ (**Figure 5B**), however, the percent of DEGs involved in these categories in ‘HR’ were significantly greater than that in ‘HS’.

KEGG analysis was performed to identify the potential biological pathways of genes represented in the transcriptome after heat stress in jujube. Results showed that 5, 7, and 7 pathways were significantly enriched for DEGs in HR1 vs HR0, HR3 vs HR0, HR5 vs HR0, respectively, and 4, 5, 8 pathways were significantly enriched in HS1 vs HS0, HS3 vs HS0, HS5 vs HS0, respectively (**S5 Table**). Carbon metabolism (ko01200) and carbon fixation in photosynthetic organisms (ko00710) were significantly enriched in 'HR' and 'HS' after 1, 3, 5d of heat stress, indicating that the two pathways possibly played an important role in heat response of jujube seedlings. Additionally, heat shock protein genes belonging to the protein processing in endoplasmic reticulum (ko04141) pathway were significantly enriched in HR1 vs HR0, suggesting that 'HR' can sharply accumulate heat shock proteins to improve the heat tolerance of the plants when suffering from heat stress, which was consistent with the previous study on spinach[21].

HSPs Related to Heat Stress

It is well known that HSP family exert crucial roles in responding to heat stress and could be immediately induced by high temperatures [38,39,40]. The HSPs can be divided into five structurally distinct classes, including: HSP100, HSP90, HSP70, HSP60, and small HSPs (sHSPs) [41]. Small HSPs, which are especially abundant in plants, plays important roles in abiotic stress tolerance and are found to be in great abundance and diversity[42]. Overexpression of a small heat shock protein, CaHSP16.4, of pepper (*Capsicum annuum* L.) enhances tolerance to heat stress in *Arabidopsis thaliana*[43]. Wang et al.[44]reported that the expression of protein HSP21 was induced by heat stress in grapevine leaves, and decreased in the control during the recovery. Overexpression of *EaHSP70* gene from *Erianthus arundinaceus* confers drought and salinity tolerance in sugarcane[45]. In this study, four *HSP90s*, two *HSP70s*, and eighteen *sHSPs* were identified as candidate genes in different HSP families, among which, *sHSPs* including *HSP26.5*, *HSP22.0*, *HSP18.5*, *HSP17.4*, *HSP17.1* and *HSP16.9*. All *HSPs* (except LOC107413527) were significantly up-regulated in both 'HR' and 'HS' jujube cultivar under heat stress (**S6 Table**). In addition, one DEG (LOC107420074) was up-regulated only in 'HR' and one DEG (LOC107429130) was only up-regulated in 'HS'. Two *sHSPs* (LOC107407995, LOC107410965) were sharply responsive to heat stress in 'HR' at 1 d and the expression levels increased after 3, 5 d of heat stress, while the two *sHSPs* were identified to be up-regulated in 'HS' only after 5 d of heat stress.

Transcription Factors Involved in Heat Stress

Previous studies had reported that transcription factors (TF) play crucial roles in regulating the expression of specific downstream genes in abiotic stresses, such as high temperatures, cold, high salinity, and drought [46,47]. In our present study, 86 and 52 DEGs were respectively identified to be involved in eight TF families in 'HR' and 'HS' during the heat stress (**S7 Table**). These TF families include *ERF*, *WRKY*, *MYB*, *NAC*, *DERB*, *bHLH*, *HSF* and *C2H2* (Table 2). There were 21 common transcription factors in HR1d vs.HR0d, HR3d vs.HR0d, and HR5d vs.HR0d, among which, 16 genes encoding four *ERFs* (LOC107432807, LOC107404986, LOC107408332, LOC107417413), six *NACs* (LOC107419222, LOC107421097, LOC107428947, LOC107435293, LOC107406551, and Ziziphus_jujuba_newGene_7074), one *MYB* (LOC107426233), one *bHLH* (LOC107421731), two *zinc finger proteins* (LOC107417576, LOC107424717), two *HSFs* (LOC107431837, LOC107429964) were up-regulated and 5 genes encoding two *ERFs* (LOC107435298, LOC107415044), one *WRKY* (LOC107432867), one *bHLH* (LOC107432409), one *zinc finger protein* (LOC107416167) were down-regulated in 'HR' at all the heat stress time points. In addition, 15 common transcription factor genes encoding six *ERFs* (LOC107404986, LOC107405213, LOC107408332, LOC107424539, LOC107435298, LOC107415044), two *NACs* (LOC107406551, LOC107416163), one *MYB* (LOC107412845), two *bHLHs* (LOC107421731, LOC107432409), two *zinc finger proteins* (LOC107417576, LOC107424717), two *HSFs* (LOC107431837, LOC107429964) were identified in HS1d vs. HS0d, HS3d vs. HS0d, and HS5d vs. HS0d. All of the comparisons shared 11 transcription

factors, including 8 up-regulated genes and 3 down-regulated genes at all the stages of heat stress in 'HR' and 'HS'. Therefore, these TFs may play important roles in response to heat stress in jujube.

HSFs have been shown to be involved in heat stress response[48]. Most plant HSFs are mostly regulated by heat stress. In pepper (*Capsicum annum* L.), *HSFA3* was more induced by heat stress in the heat-susceptible cultivar 'S590', and *HSFA2* was upregulated in the heat-tolerant cultivar 'R597' [22]. Yan et al. [21] reported that fourteen *HSFs* were induced in spinach leaves during the heat treatment. In our study, we found one heat stress transcription factor C1 (LOC107431837) were up-regulated in both 'HR' and 'HS' during the heat treatment, however, *HSFC1* (LOC107431837) had a 2.39, 3.47, 3.54 fold changes after 1, 3, 5d of heat stress in 'HR', but 1.57, 1.89, 1.94 fold changes in 'HS', respectively, which showed an dramatic increase in expression in 'HR' than that observed in 'HS'.

Table 2. The number of DEGs identified as transcription factors in jujube leaves under heat stress

Category	Heat-Resistant, HR						Total	Heat-Sensitive, HS						Total
	1d		3d		5d			1d		3d		5d		
	Up	Down	Up	Down	Up	Down		Up	Down	Up	Down	Up	Down	
ERF	6	3	8	8	5	9	19	4	3	9	3	5	5	15
WRKY	4	1	0	1	1	3	7	0	0	0	0	1	1	2
NAC	7	0	10	0	9	0	10	6	0	4	0	7	1	10
MYB	6	1	4	5	5	6	17	0	2	1	2	0	4	5
DREB	0	0	0	1	1	1	2	0	0	0	1	0	1	1
bHLH	1	1	1	2	1	3	5	1	2	1	1	1	3	4
C2H2	2	2	7	7	9	10	20	3	0	3	1	5	3	10
HSF	3	0	6	0	3	0	6	2	0	3	0	4	0	5

Expression Analysis of Ubiquitin-Protein Ligase Under Heat Stress

The ubiquitin-proteasome system (UPS) is a central regulator that controls plant response and adaptation to environmental stresses[49]. The UPS is composed of three enzymes: E1 (ubiquitin activating enzymes), E2 (ubiquitin conjugating enzymes), and E3 (ubiquitin ligases)[50]. Overexpression of a rice RING finger E3 ligase, *OsHCI1*, confers enhanced heat tolerance in *Arabidopsis*[51]. Morimoto et al.[52] found that BPM-CUL3 E3 ligase could modulate the heat stress response and prevent an adverse effect of excess DREB2A on plant growth in *Arabidopsis*. In our study, we identified twenty ubiquitin-protein ligase genes the expression patterns were shown in **Figure 6**. Four genes encoding ubiquitin-protein ligase (LOC107414605, LOC107432264, LOC107418004, LOC107428857) were all down-regulated in both 'HR' and 'HS' under heat stress, suggesting that these four genes were repressed when jujube suffered from heat stress. In addition, the expression of 15 genes encoding ubiquitin-protein ligase (LOC107428626, LOC107424969, LOC107413314, LOC107413313, LOC107422277, LOC107426882, LOC107420280, LOC107415107, LOC107432321, LOC107419944, LOC107404052, LOC107431904, LOC107411111, LOC107431883, LOC107403709) were all higher in 'HR' than in 'HS' at 3, 5d, indicating that the high expression of these ubiquitin-protein ligases may be the reason for the heat tolerance of 'HR' to high temperature.

Validation of DEGs by qRT-PCR

To confirm the validation of the RNA-seq data, nine DEGs were selected for real-time PCR analysis and they included HSP16.9 (LOC107413804), HSP70 (LOC107423197), HSP83-like (LOC107430207), HSFc-1(LOC107431837), HSP transcription factor (LOC107429964), bHLH48-like(LOC107432409), E3 ubiquitin-protein ligase RHA2A (LOC107413313), E3 ubiquitin-protein ligase SINAT2 (LOC107415107), and E3 ubiquitin-protein ligase RHF1A (LOC107426882). As shown in **Figure 7**, the expression patterns of both qRT-PCR and RNA-seq data were highly consistent.

Conclusions

In this study, transcriptome analysis was performed on heat-resistant cultivar 'HR' and heat-sensitive cultivar 'HS' after 0,1, 3, and 5d of heat stress. Totally, 1880, 4350, 5435 differentially expressed genes were identified in 'HR', and 2390, 2998, 4064 genes were detected in 'HS' after heat stress for 1, 3 and 5d, respectively. Some DEGs related to heat shock proteins, transcription factors, and ubiquitin-protein ligase genes were identified during heat stress. Therefore, this study not only provided a basis for further understanding of the molecular mechanism on heat tolerance of jujube plants, but also exerted valuable and useful genes involved in heat stress, which would be helpful for the genetic improvement of heat tolerance in jujube breeding.

Supporting information

S1 Table. Primers used in the study for quantitative RT-PCR.
(XLSX)

S2 Table. Summary of the sequencing results.
(XLSX)

S3 Table. List of DEGs between 'HR' and 'HS'.
(XLSX)

S4 Table. GO classification of DEGs between 'HR' and 'HS'.
(XLSX)

S5 Table. Pathway analysis of DEGs in different comparisons.
(XLSX)

S6 Table. Expression of DEGs related to heat shock proteins.
(XLSX)

S7 Table. Transcription Factors Involved in Heat Stress.
(XLSX)

Figures and Figure legends

Figure 1. Ultrastructure observation and physiological analyses of heat-resistant 'HR' and heat-sensitive 'HS' jujube cultivars subjected to heat stress. (A) Characteristic structures in non-stressed (0 d) and heat stressed leaves observed by transmission electron microscopy. CW cell membrane, NM nucleus membrane, N nucleus, Nu nucleolus, Ch chloroplast, M mitochondrion, Sg starch grain, Og osmiophilic granule. The magnification of these pictures is × 2.5k. The bars represent 5 μm. (B) Changes in electrolyte leakage, (C) MDA content, and (D) proline content in leaves of two jujube cultivars exposed to 45°C for 0, 1, 3, and 5 d, respectively. Error bars are ± SD of the mean (n = 3). Samples were collected from nine plants for each replicate. Duncan's multiple range test (P < 0.05) was used to determine significant differences between treatments. These differences are shown by the letters above the bars.

Figure 2. Overview of the Illumina transcriptome sequencing. (A) Gene expression levels of all the 24 libraries. HR represents the heat resistant jujube cultivar, HS represents the heat sensitive jujube cultivar. (B) Heatmap clustering showing the sample correlation analysis of the 24 sequenced samples. The number 1, 2, 3 and 4 represent the 4 Clusters of samples.

Figure 3. Venn diagrams of the overlapped DEGs identified in 'HR' and 'HS' under heat stress

Figure 4. Venn diagrams of up- and down-regulated genes of different heat stress time points between 'HR' and 'HS' after 0, 1, 3, 5d of heat stress

Figure 5. GO enrichment analysis of the DEGs between the two cultivars after different time heat stress. (A) GO enrichment analysis of 'HR' after 1, 3 and 5d of heat stress compared with 0d. (B) GO enrichment analysis of 'HS' after 1, 3 and 5d of heat stress compared with 0d.

Figure 6. Expression patterns of twenty ubiquitin-protein ligase genes in 'HR' and 'HS' after 0, 1, 3, 5d of heat stress. Gene expression level was measured using the FPKM method. Horizontal axes represent gene ID, and vertical axes represent 0, 1, 3, and 5 d (from left to right) of heat stress in HR and HS. Heat map shows the log_2 FPKM values ranging from green (low expression) to red (high expression).

Figure 7. Expression analysis of nine selected DEGs in 'HR' and 'HS' after 0, 1, 3, 5d of heat stress. (A) qRT-PCR results of nine DEGs. The error bars represent SD (n=3). (B) Heat map shows the log_2 FPKM values at different heat stress time points ranging from green (low expression) to red (high expression).

Acknowledgments

We would like to thank Biomarker Technologies Co., Ltd., Beijing, China for technical help.

Author contributions

Juan Jin, Lei Yang, Dingyu Fan, Xuxin Liu and Qing Hao
Conceptualization: Juan Jin, Xuxin Liu, Qing Hao.
Data curation: Juan Jin, Lei Yang, Dingyu Fan.
Formal analysis: Juan Jin.
Funding acquisition: Juan Jin.
Methodology: Lei Yang, Dingyu Fan.
Supervision: Qing Hao.
Writing ± original draft: Juan Jin.
Writing ± review & editing: Juan Jin.

References

1. Zhu JK. Abiotic Stress Signaling and Responses in Plants. Cell. 2016;167: 313–324. doi:10.1016/j.cell.2016.08.029

2. Bita CE, Gerats T. Plant tolerance to high temperature in a changing environment: Scientific fundamentals and production of heat stress-tolerant crops. Front Plant Sci. 2013;4: 273. doi:10.3389/fpls.2013.00273

3. Balfagón D, Zandalinas SI, Gómez-Cadenas A. High temperatures change the perspective: Integrating hormonal responses in citrus plants under co-occurring abiotic stress conditions. Physiol Plant. 2019;165: 183–197. doi:10.1111/ppl.12815

4. Zandalinas SI, Mittler R, Balfagón D, Arbona V, Gómez-Cadenas A. Plant adaptations to the combination of drought and high temperatures. Physiol Plant. 2018;162: 2–12. doi:10.1111/ppl.12540

5. Awasthi R, Bhandari K, Nayyar H. Temperature stress and redox homeostasis in agricultural crops. Front Environ Sci. 2015;3: 1–24. doi:10.3389/fenvs.2015.00011

6. Mittler R, Finka A, Goloubinoff P. How do plants feel the heat? Trends Biochem Sci. 2012;37: 118–125. doi:10.1016/j.tibs.2011.11.007

7. Almeselmani M, Deshmukh PS, Sairam RK, Kushwaha SR, Singh TP. Protective role of

antioxidant enzymes under high temperature stress. Plant Sci. 2006;171: 382–388. doi:10.1016/j.plantsci.2006.04.009

8. Hasanuzzaman M, Nahar K, Alam MM, Roychowdhury R, Fujita M. Physiological, biochemical, and molecular mechanisms of heat stress tolerance in plants. Int J Mol Sci. 2013;14: 9643–9684. doi:10.3390/ijms14059643

9. Wang W, Vinocur B, Shoseyov O, Altman A. Role of plant heat-shock proteins and molecular chaperones in the abiotic stress response. Trends in Plant Science. 2004. pp. 244–252. doi:10.1016/j.tplants.2004.03.006

10. Ohama N, Sato H, Shinozaki K, Yamaguchi-Shinozaki K. Transcriptional Regulatory Network of Plant Heat Stress Response. Trends Plant Sci. 2017;22: 53–65. doi:10.1016/j.tplants.2016.08.015

11. Kotak S, Larkindale J, Lee U, von Koskull-Döring P, Vierling E, Scharf KD. Complexity of the heat stress response in plants. Curr Opin Plant Biol. 2007;10: 310–316. doi:10.1016/j.pbi.2007.04.011

12. Baxter A, Mittler R, Suzuki N. ROS as key players in plant stress signalling. J Exp Bot. 2014;65: 1229–1240. doi:10.1093/jxb/ert375

13. Qu AL, Ding YF, Jiang Q, Zhu C. Molecular mechanisms of the plant heat stress response. Biochem Biophys Res Commun. 2013;432: 203–207. doi:10.1016/j.bbrc.2013.01.104

14. Zhong L, Zhou W, Wang H, Ding S, Lu Q, Wen X, et al. Chloroplast small heat shock protein HSP21 interacts with plastid nucleoid protein pTAC5 and is essential for chloroplast development in arabidopsis under heat stress. Plant Cell. 2013;25: 2925–2943. doi:10.1105/tpc.113.111229

15. Chakraborty K, Bishi SK, Singh AL, Zala P V., Mahatma MK, Kalariya KA, et al. Rapid induction of small heat shock proteins improves physiological adaptation to high temperature stress in peanut. J Agron Crop Sci. 2018;204: 285–297. doi:10.1111/jac.12260

16. Zhou Y, Yang P, Cui F, Zhang F, Luo X, Xie J. Transcriptome analysis of salt stress responsiveness in the seedlings of dongxiang wild rice (Oryza rufipogon Griff.). PLoS One. 2016;11: 1–25. doi:10.1371/journal.pone.0146242

17. Guo R, Wang X, Han X, Li W, Liu T, Chen B, et al. Comparative transcriptome analyses revealed different heat stress responses in high- and low-GS Brassica alboglabra sprouts. BMC Genomics. 2019;20: 1–17. doi:10.1186/s12864-019-5652-y

18. Wang W, Lin Y, Teng F, Ji D, Xu Y, Chen C, et al. Comparative transcriptome analysis between heat-tolerant and sensitive Pyropia haitanensis strains in response to high temperature stress. Algal Res. 2018;29: 104–112. doi:10.1016/j.algal.2017.11.026

19. Wang A, Hu J, Huang X, Li X, Zhou G, Yan Z. Comparative Transcriptome Analysis Reveals

Heat-Responsive Genes in Chinese Cabbage (Brassica rapa ssp. chinensis). Front Plant Sci. 2016;7: 1–14. doi:10.3389/fpls.2016.00939

20. Shi J, Yan B, Lou X, Ma H, Ruan S. Comparative transcriptome analysis reveals the transcriptional alterations in heat-resistant and heat-sensitive sweet maize (Zea mays L.) varieties under heat stress. BMC Plant Biol. 2017;17: 1–10. doi:10.1186/s12870-017-0973-y

21. Yan J, Yu L, Xuan J, Lu Y, Lu S, Zhu W. De novo transcriptome sequencing and gene expression profiling of spinach (Spinacia oleracea L.) leaves under heat stress. Sci Rep. 2016;6: 1–10. doi:10.1038/srep19473

22. Li T, Xu X, Li Y, Wang H, Li Z, Li Z. Comparative transcriptome analysis reveals differential transcription in heat-susceptible and heat-tolerant pepper (Capsicum annum L.) cultivars under heat stress. J Plant Biol. 2015;58: 411–424. doi:10.1007/s12374-015-0423-z

23. Yao S. Past, present, and future of jujubes-Chinese dates in the United States. HortScience. 2013;48: 672–680.

24. Bai T, Zhang N, Chen Y, Mercatoris B. Assessing the performance of the WOFOST model in simulating jujube fruit tree growth under different irrigation regimes. Sustain. 2019;11. doi:10.3390/su11051466

25. Yang G, Rhodes D, Joly RJ. Effects of high temperature on membrane stability and chlorophyll fluorescence in glycinebetaine-deficient and glycinebetaine- containing maize lines. Aust J Plant Physiol. 1996;23: 437–443. doi:10.1071/PP9960437

26. Heath, RL PL. Photoperoxidation in isolated Chloroplasts of Fatty Acid Peroxidation chlorophyll. Arch Biochem biophisics. 1968;126: 189–198.

27. Singh IN, Paleg IG, Aspinall D. Stress metabolism I. Nitrogen metabolism and growth in the barley plant during water stress. Aust J Biol Sci. 1973;26: 45–56. doi:10.1071/BI9730045

28. Liu MJ, Zhao J, Cai Q Le, Liu GC, Wang JR, Zhao ZH, et al. The complex jujube genome provides insights into fruit tree biology. Nat Commun. 2014;5: 1–12. doi:10.1038/ncomms6315

29. Langmead B, Salzberg SL. Fast gapped-read alignment with Bowtie 2. Nat Methods. 2012;9: 357–359. doi:10.1038/nmeth.1923

30. Kim D, Pertea G, Trapnell C, Pimentel H, Kelley R, Salzberg SL. TopHat2: Accurate alignment of transcriptomes in the presence of insertions, deletions and gene fusions. Genome Biol. 2013;14: 1–13. doi:10.1186/gb-2013-14-4-r36

31. Li H, Handsaker B, Wysoker A, Fennell T, Ruan J, Homer N, et al. The Sequence Alignment/Map format and SAMtools. Bioinformatics. 2009;25: 2078–2079. doi:10.1093/bioinformatics/btp352

32. Trapnell C, Williams BA, Pertea G, Mortazavi A, Kwan G, Van Baren MJ, et al. Transcript assembly and quantification by RNA-Seq reveals unannotated transcripts and isoform switching during cell differentiation. Nat Biotechnol. 2010;28: 511–515. doi:10.1038/nbt.1621

33. Wang L, Feng Z, Wang X, Wang X, Zhang X. DEGseq: An R package for identifying differentially expressed genes from RNA-seq data. Bioinformatics. 2009;26: 136–138. doi:10.1093/bioinformatics/btp612

34. Young MD, Wakefield MJ, Smyth GK, Oshlack A. Gene ontology analysis for RNA-seq: accounting for selection bias. Genome Biol. 2010;11. doi:10.1186/gb-2010-11-2-r14

35. Mao X, Cai T, Olyarchuk JG, Wei L. Automated genome annotation and pathway identification using the KEGG Orthology (KO) as a controlled vocabulary. Bioinformatics. 2005;21: 3787–3793. doi:10.1093/bioinformatics/bti430

36. Bu J, Zhao J, Liu ML. Expression stabilities of candidate reference genes for RT-qPCR in chinese jujube (Ziziphus jujuba Mill.) under a variety of conditions. PLoS One. 2016;11: 1–11. doi:10.1371/journal.pone.0154212

37. Livak KJ, Schmittgen TD. Analysis of relative gene expression data using real-time quantitative PCR and the 2-ΔΔCT method. Methods. 2001;25: 402–408. doi:10.1006/meth.2001.1262

38. Zhang X, Li J, Liu A, Zou J, Zhou X, Xiang J, et al. Expression Profile in Rice Panicle: Insights into Heat Response Mechanism at Reproductive Stage. PLoS One. 2012;7. doi:10.1371/journal.pone.0049652

39. Liu GT, Wang JF, Cramer G, Dai ZW, Duan W, Xu HG, et al. Transcriptomic analysis of grape (Vitis vinifera L.) leaves during and after recovery from heat stress. BMC Plant Biol. 2012;12. doi:10.1186/1471-2229-12-174

40. Wang M, Jiang B, Liu W, Lin Y, Liang Z, He X, et al. Transcriptome Analyses Provide Novel Insights into Heat Stress Responses in Chieh-Qua (Benincasa hispida Cogn. var. Chieh-Qua How). Int J Mol Sci. 2019;20: 883. doi:10.3390/ijms20040883

41. Huang B, Xu C. Identification and characterization of proteins associated with plant tolerance to heat stress. J Integr Plant Biol. 2008;50: 1230–1237. doi:10.1111/j.1744-7909.2008.00735.x

42. Jiang C, Xu J, Zhang H, Zhang X, Shi J, Li M, et al. A cytosolic class i small heat shock protein, RcHSP17.8, of Rosa chinensis confers resistance to a variety of stresses to Escherichia coli, yeast and Arabidopsis thaliana. Plant, Cell Environ. 2009;32: 1046–1059. doi:10.1111/j.1365-3040.2009.01987.x

43. Huang LJ, Cheng GX, Khan A, Wei AM, Yu QH, Yang SB, et al. CaHSP16.4, a small heat shock protein gene in pepper, is involved in heat and drought tolerance. Protoplasma. 2019;256: 39–51. doi:10.1007/s00709-018-1280-7

44. Wang LJ, Fan L, Loescher W, Duan W, Liu GJ, Cheng JS, et al. Salicylic acid alleviates decreases in photosynthesis under heat stress and accelerates recovery in grapevine leaves. BMC Plant Biol. 2010;10. doi:10.1186/1471-2229-10-34

45. Augustine SM, Narayan JA, Syamaladevi DP, Appunu C, Chakravarthi M, Ravichandran V, et al. Erianthus arundinaceus HSP70 (EaHSP70) overexpression increases drought and salinity tolerance in sugarcane (Saccharum spp. hybrid). Plant Sci. 2015;232: 23–34. doi:10.1016/j.plantsci.2014.12.012

46. Wahid A, Gelani S, Ashraf M, Foolad MR. Heat tolerance in plants: An overview. Environmental and Experimental Botany. 2007. pp. 199–223. doi:10.1016/j.envexpbot.2007.05.011

47. Golldack D, Lüking I, Yang O. Plant tolerance to drought and salinity: Stress regulating transcription factors and their functional significance in the cellular transcriptional network. Plant Cell Rep. 2011;30: 1383–1391. doi:10.1007/s00299-011-1068-0

48. Pirkkala L, Nykänen P, Sistonen L. Roles of the heat shock transcription factors in regulation of the heat shock response and beyond. FASEB J. 2001;15: 1118–1131. doi:10.1096/fj00-0294rev

49. Serrano I, Campos L, Rivas S. Roles of E3 ubiquitin-ligases in nuclear protein homeostasis during plant stress responses. Front Plant Sci. 2018;9: 1–7. doi:10.3389/fpls.2018.00139

50. Stone SL. The role of ubiquitin and the 26S proteasome in plant abiotic stress signaling. Front Plant Sci. 2014;5: 1–10. doi:10.3389/fpls.2014.00135

51. Lim SD, Cho HY, Park YC, Ham DJ, Lee JK, Jang CS. The rice RING finger E3 ligase, OsHCI1, drives nuclear export of multiple substrate proteins and its heterogeneous overexpression enhances acquired thermotolerance. J Exp Bot. 2013;64: 2899–2914. doi:10.1093/jxb/ert143

52. Morimoto K, Ohama N, Kidokoro S, Mizoi J, Takahashi F, Todaka D, et al. BPM-CUL3 E3 ligase modulates thermotolerance by facilitating negative regulatory domain-mediated degradation of DREB2A in Arabidopsis. Proc Natl Acad Sci U S A. 2017;114: E8528–E8536. doi:10.1073/pnas.1704189114

Figure 1. Ultrastructure observation and physiological analyses of heat-resistant ‘HR’ and heat-sensitive ‘HS’ jujube cultivars subjected to heat stress

Click here to access/download;Figure;Figure 1.jpg

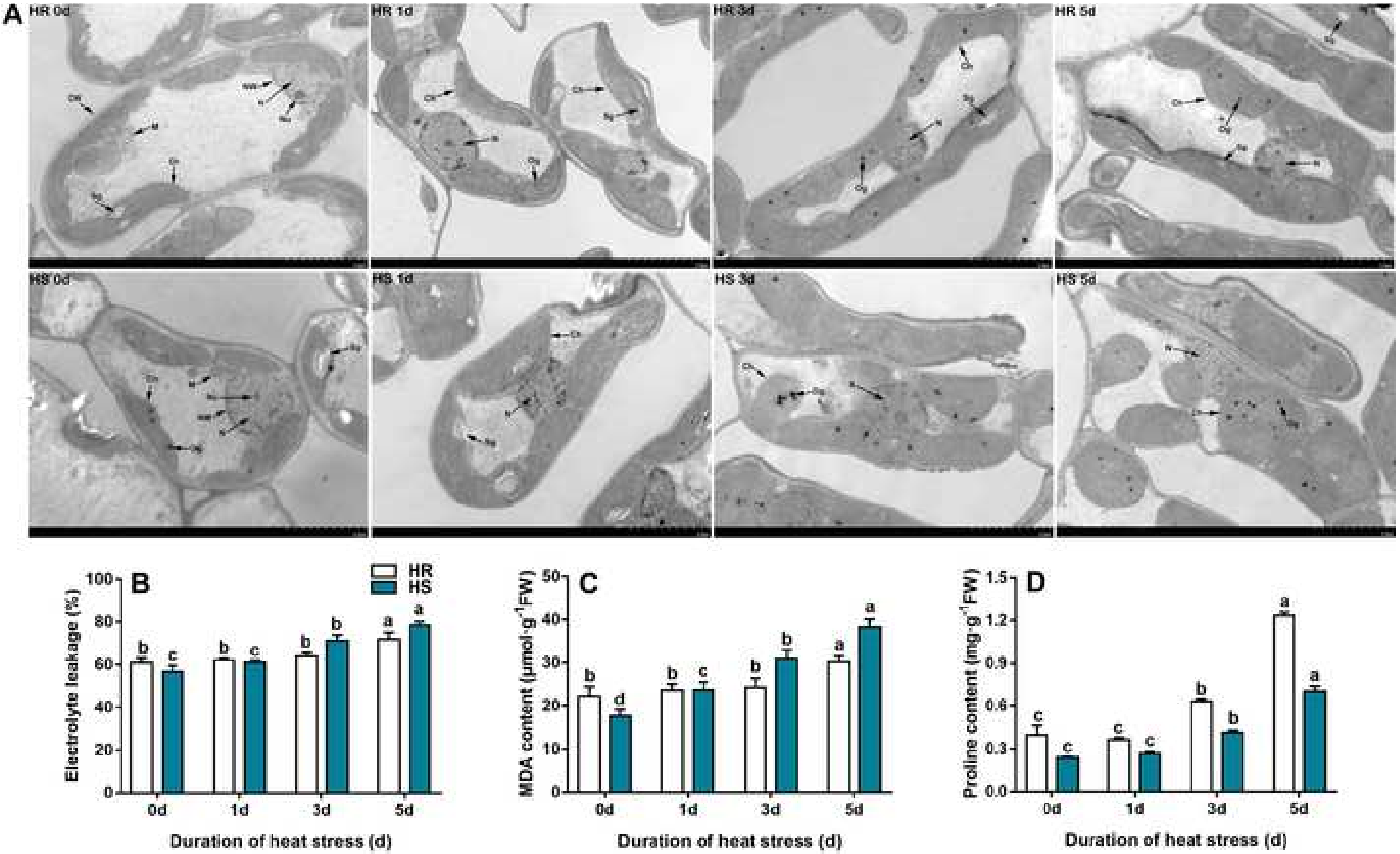

Figure 2. Overview of the Illumina transcriptome sequencing

Click here to access/download;Figure;Figure 2.jpg

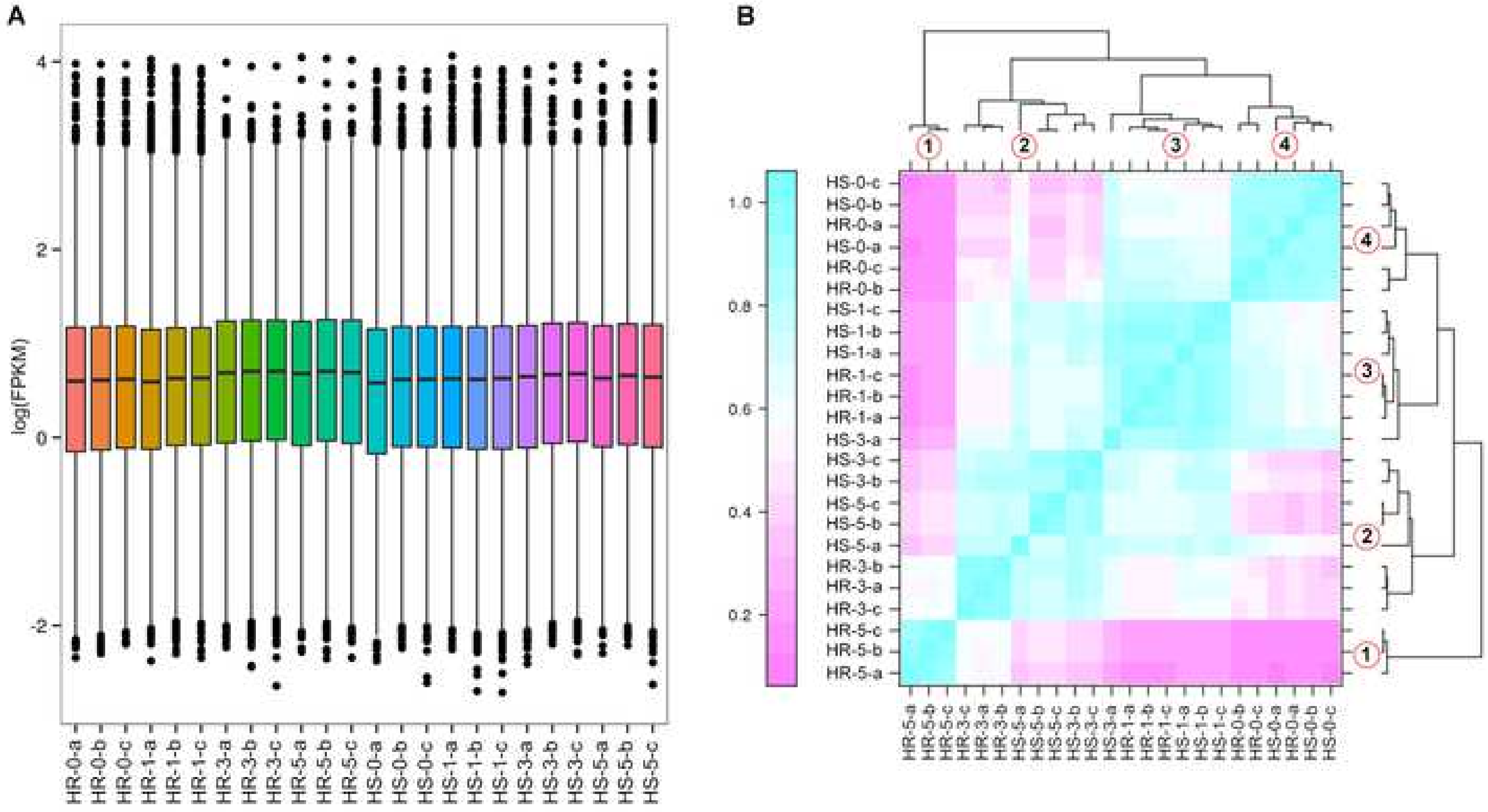

Figure 3. Venn diagrams of the overlapped DEGs identified in ‘HR’ and ‘HS’ under heat stress

Click here to access/download;Figure;Figure 3.jpg

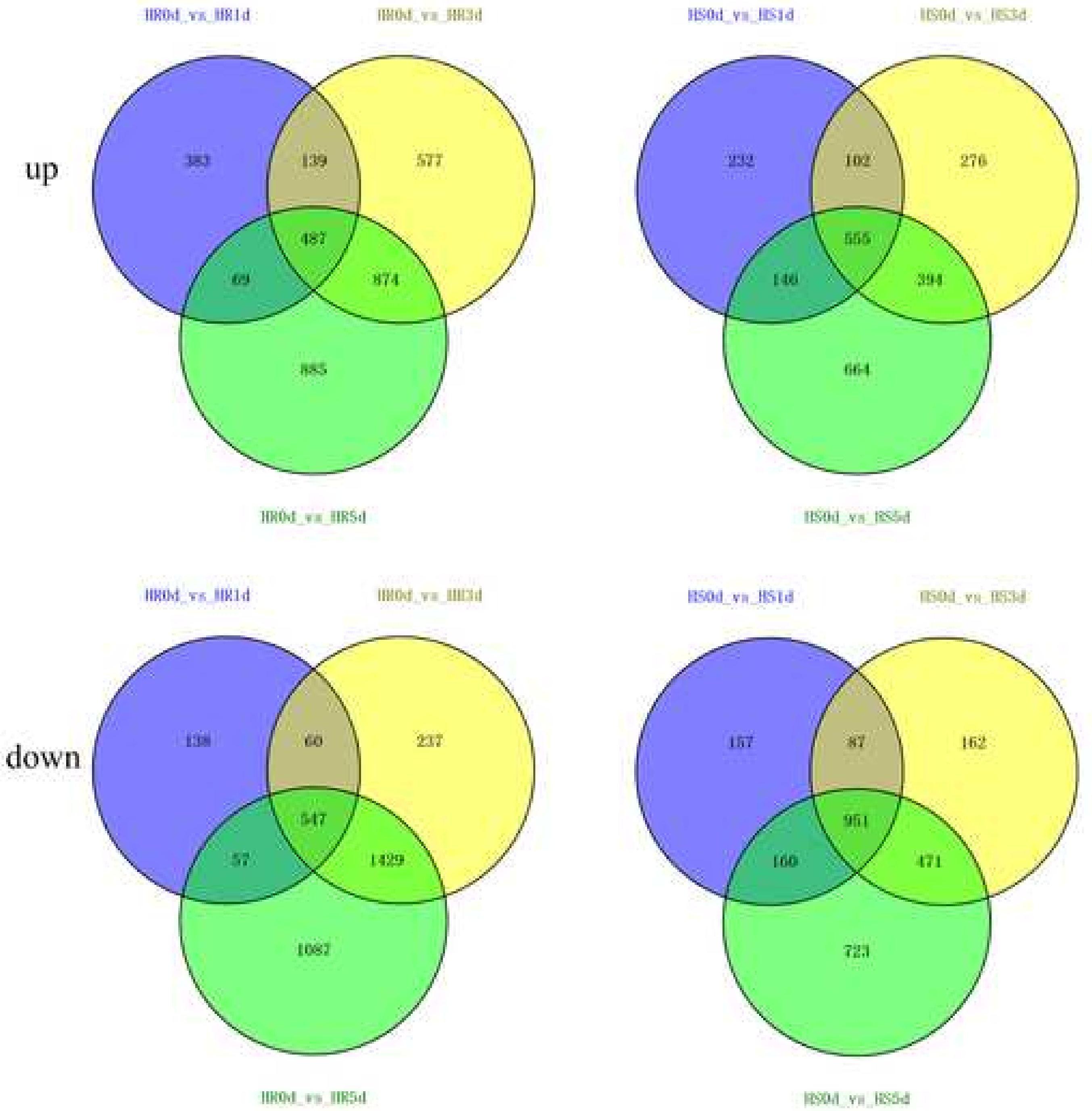

Figure 4. Venn diagrams of up- and down-regulated genes of different heat stress time points between ‘HR’ and ‘HS’ after 0, 1, 3, 5d of heat stress

Click here to access/download;Figure;Figure 4.jpg

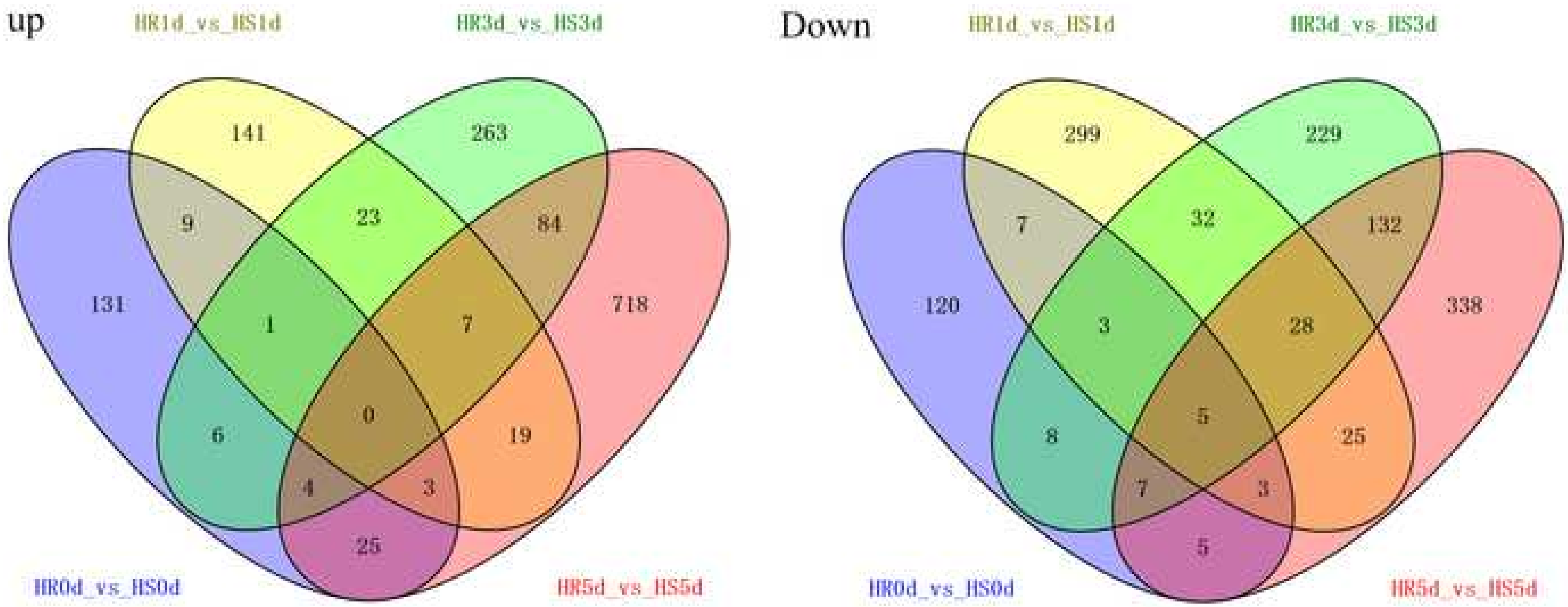

Figure 5. GO enrichment analysis of the DEGs between the two cultivars after different time heat stress

Click here to access/download;Figure;Figure 5.jpg

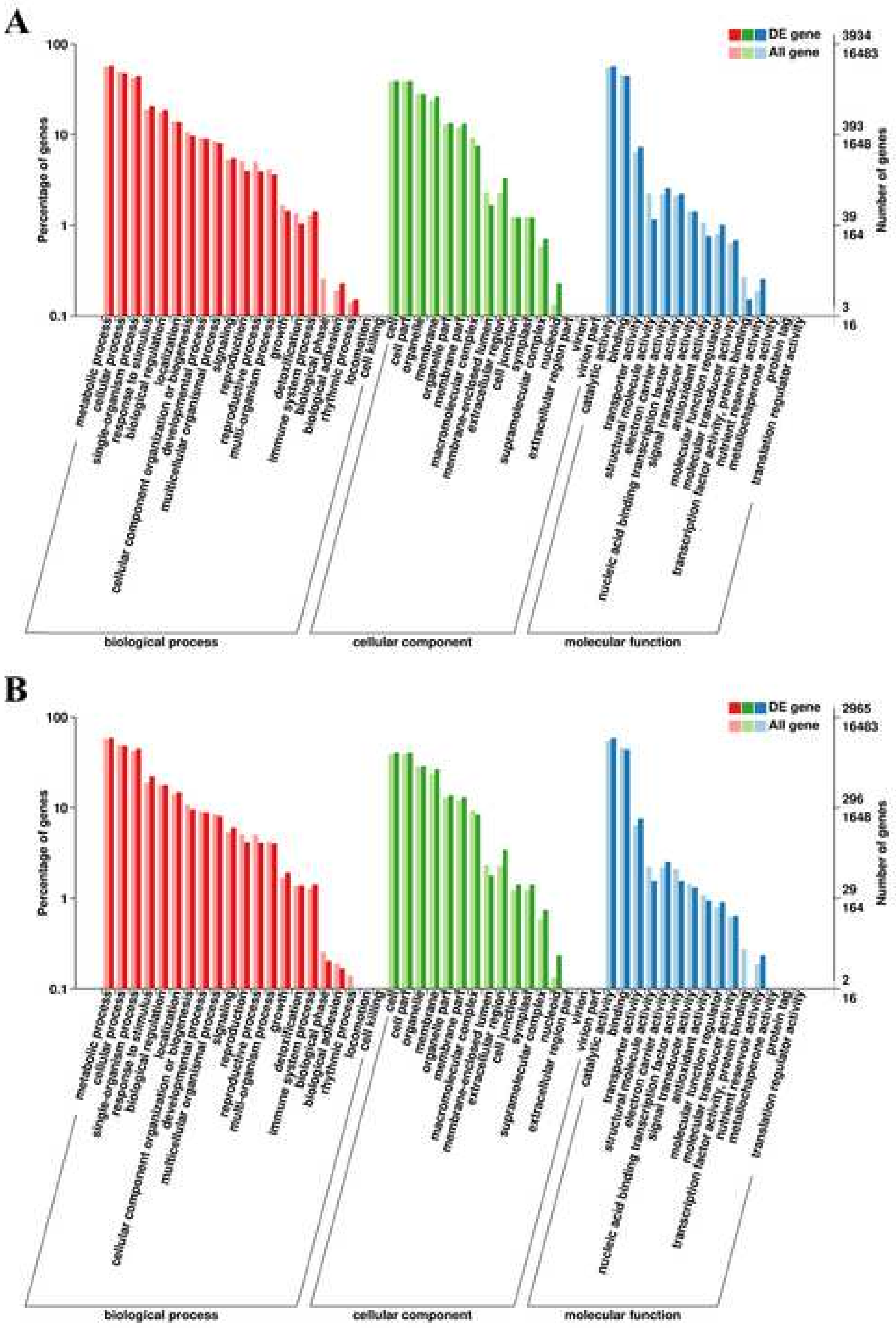

Figure 6. Expression patterns of twenty ubiquitin-protein ligase genes in 'HR' and 'HS' after 0, 1, 3, 5d of heat stress

Click here to access/download;Figure;Figure 6.jpg

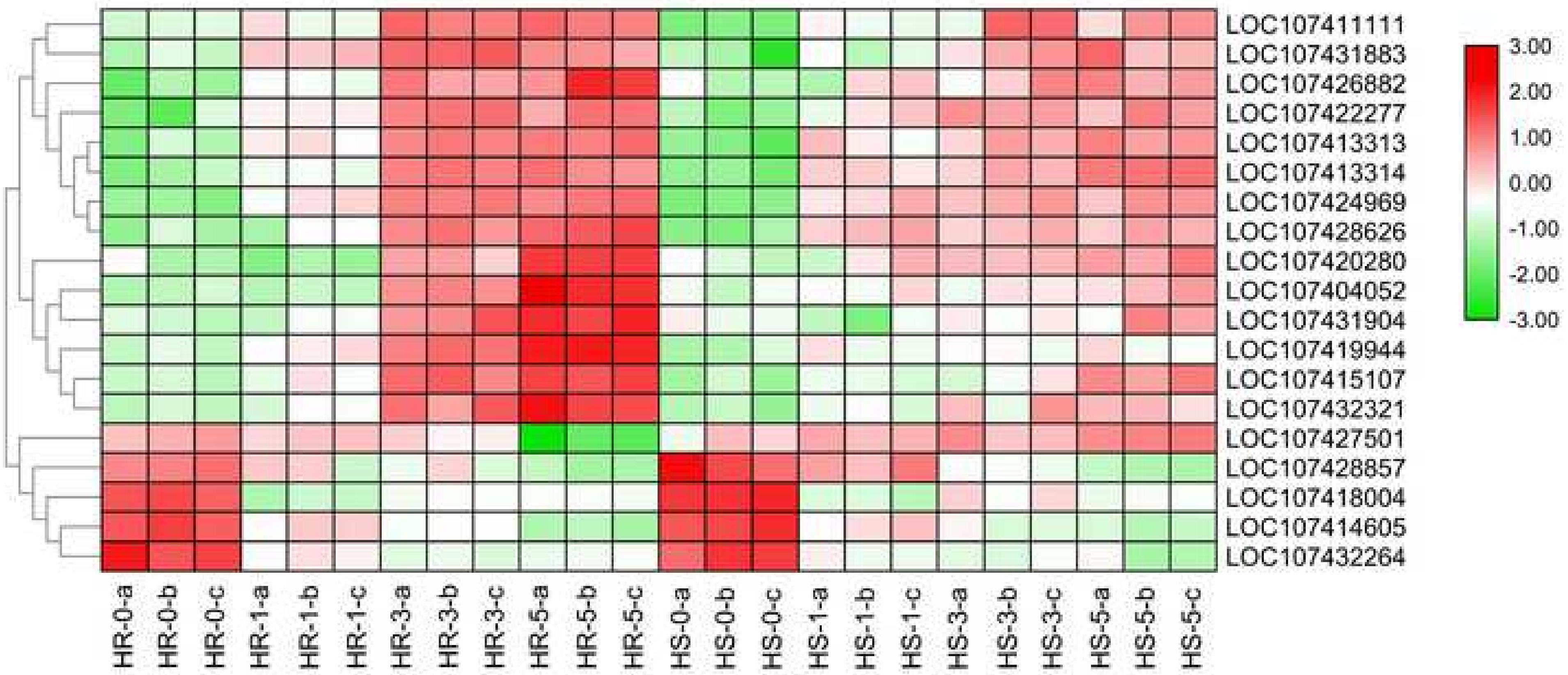

Figure 7. Expression analysis of nine selected DEGs in 'HR' and 'HS' after 0, 1, 3, 5d of heat stress

Click here to access/download;Figure;Figure 7.jpg

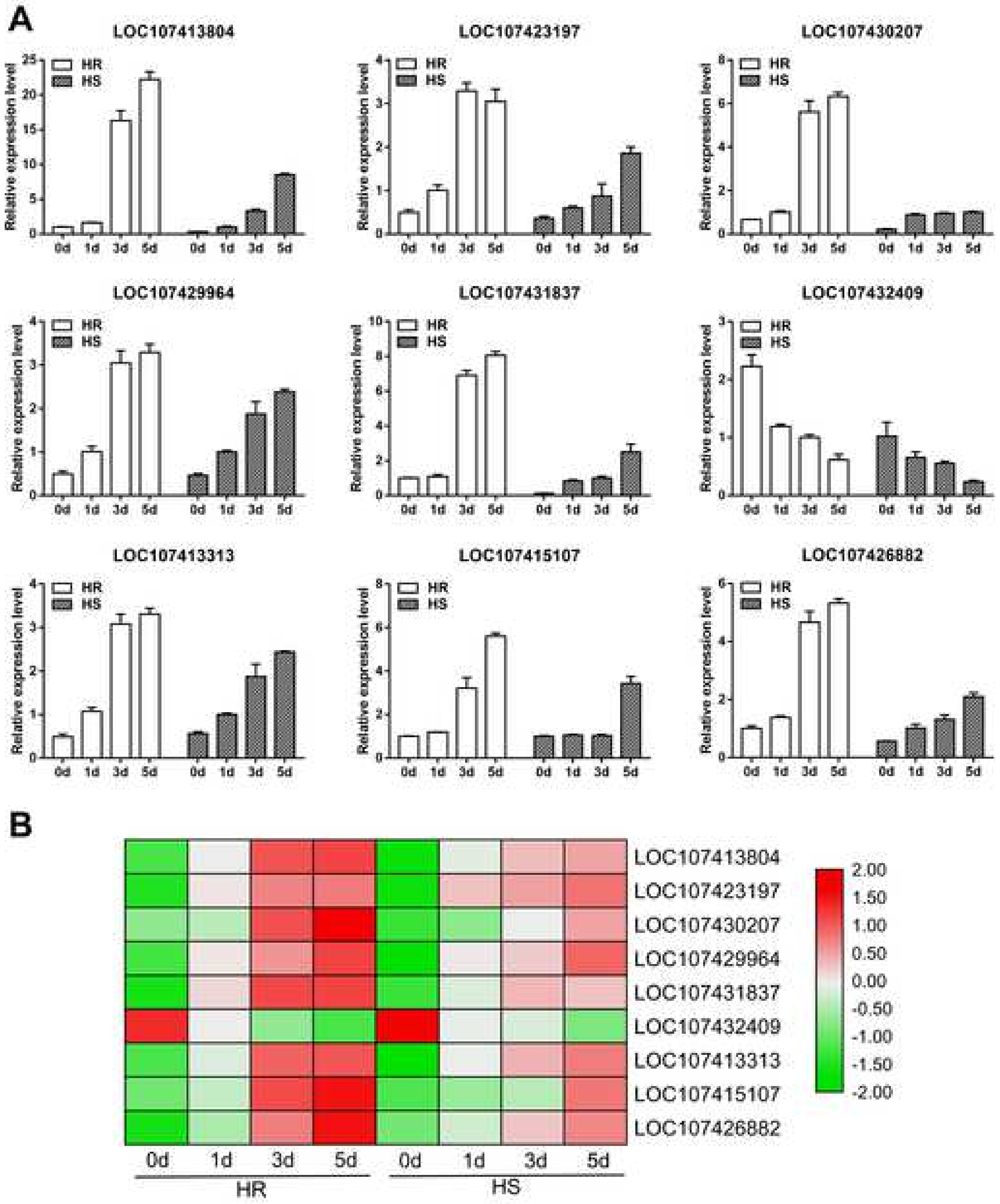

S1 Table. Primers used in the study for quantitative RT-PCR.

Click here to access/download
Supporting Information
S1 Primers used in the study for quantitative RT-PCR.xls

S2 Table. Summary of the sequencing results.

Click here to access/download
Supporting Information
S2 Summary of the sequencing results.xls

S3 Table. List of DEGs between ‘HR’ and ‘HS’.

Click here to access/download
Supporting Information
S3 List of DEGs between HR and HS.xlsx

S4 Table. GO classification of DEGs between ‘HR’ and ‘HS’.

Click here to access/download
Supporting Information
S4 GO classification of DEGs between HR and HS.xlsx

S5 Table. Pathway analysis of DEGs in different comparisons.

Click here to access/download
Supporting Information
S5 Pathway analysis of DEGs in different comparisons.xlsx

S6 Table. Expression of DEGs related to heat shock proteins.

Click here to access/download
Supporting Information
S6 Expression of DEGs related to heat shock proteins.xlsx

S7 Table. Transcription Factors Involved in Heat Stress.

Click here to access/download

Supporting Information

S7 Transcription Factors Involved in Heat Stress.xlsx
