# Supplementary material for: Comparative transcriptome analysis uncovers different heat stress responses in heat-resistant and heat-sensitive jujube cultivars
Source: PLoS One. 2020 Sep 21;15(9):e0235763. doi: 10.1371/journal.pone.0235763 (PMC7505471; doi:10.1371/journal.pone.0235763)
Supplement: Attachment — Submitted filename: Response to Reviewers.docx [file pone.0235763.s009.docx]

Dear editors,

The authors are very thankful to you and the reviewers for the valuable comments and remarks regarding this manuscript. We have tried to address all comments and suggestions adequately. The requested alterations/corrections have been inserted directly into the manuscript and are also described below.

We are looking forward to having the opportunity to publish our work on your journal. Thank you for your help again!

Best regards!

**Comments from Reviewers:**

**Reviewer #1: The manuscript makes a contribution to understanding genes underlying heat stress resistance in jujube. The authors have done a good job with the RNA-Seq analysis, however, additional analysis and interpretation of the data is needed. The manuscript PDF has been marked with reviewer comments and is attached.**

1. Change to "genes are involved".

Response: "genes involved" was changed to "genes are involved" in the revised manuscript.

2. Change capital "S" to lower case "s".

Response: capital "S" was changed to lower case "s" in the revised manuscript.

3. Provide more information on "HR" and "HS". Are these different jujube cultivars?

Response: Ziziphus jujuba cv.‘HR’ and Ziziphus jujuba cv.‘HS’ were grown in the Forestry Management Station of Turpan city, Forestry and Grassland Administration of Turpan city, located in Turpan, Xinjiang province, China. ‘HR’ has been proven to be resistant to heat stress while ‘HS’ is susceptible.

4. Indicate if these are three replicate plant for each of the time points of the heat treatment. This would then be a total of 12 plants for each of HR and HS.

Response: There were a total of 180 plants for ‘HR’ and ‘HS’ seedlings, respectively. Each treatment included three biological replications for each cultivar, and each biological replicate contained 15 plants. Plant samples were collected after the treatments started.

5. Indicate if the physiological indexes were measured for replicate plants in each treatment time point and control.

Response: Three physiological indexes, including electrolyte leakage, malondialdehyde (MDA), and proline were measured 3 times (n=3) with 5 plants per replicate in each treatment time point for each cultivar in order to analyze the physiological changes of two jujube cultivars subjected to heat stress.

6. Change to "Construction of RNA."

Response: "RNA" was changed to " Construction of RNA " in the revised manuscript.

7. How do the physicochemical changes in jujube compare to other plants?

Response: Our ultrastructural observation clearly showed that the time-course heat stress exerted a more conspicuous effect on the heat-sensitive jujube cultivar ‘HS’ than heat-resistant jujube cultivar ‘HR’, the chloroplasts became more globular and the number of osmiophilic granules increased in response to heat, which were consistent with the leaf ultrastructure changes of Ara et al. 2015 in cucurbits. Moreover, we found that the starch grains of ‘HR’ were faintly visible, while the number of starch granules in ‘HS’ decreased and gradually disappeared, this phenomenon may be correlated with glucose starvation upon a decrease in photosynthetic activity under heat stress (Jin et al., 2011), confirming that ‘HS’ was susceptible to heat stress. The physiological indexes showed that heat stress increased the relative electrolyte leakage and MDA content in the two jujube cultivars, however, ‘HS’ suffered a greater degree of membrane damage than ‘HR’ (Fig.1). Similar results were reported by Kumar et al., 2012 and Li et al. (2015). Under heat stress conditions, the accumulation of proline may act as an osmoprotectant for cellular structures in response to high temperature, which might contribute to greater heat tolerance (Kumar et al., 2012). Our results showed that higher proline content was accumulated in heat-tolerant jujube cultivar ‘HR’, this finding confirmed the results of study on the heat tolerant genotypes, such as maize (Kumar et al., 2012) and sorghum (Gosavi et al.,2014), indicating that proline can be strongly correlated with the capacity of genotypes to survive heat stress. These discussions were added in the revised manuscript.

8. The text marking cellular structures on the figure was too small to read.

Response: We have changed the text font marking cellular structures on the figure in the revised manuscript.

9. Change to "RNASeq".

Response: We have changed "RNA" to "RNASeq" in the revised manuscript.

10. What were these five genes?

Response: These five genes were LOC107406551, LOC107434446, LOC107412389, Ziziphus_jujuba_newGene_4043, Ziziphus_jujuba_newGene_7074. These results were added in the revised manuscript.

11. The GO analysis is presented as bar graphs of DEG and All genes. The authors should use a statistical determination of the significance of GO term enrichment in for DEG. Software such as topGO implemented in R can provide such an analysis.

Response: We have used a statistical determination of the significance of GO term enrichment in for DEG and be presented as a graph (Figure 5) in the revised manuscript.

12. It is recommended that the KEGG analysis be presented as a bar graph or table of KEGG terms and their corrected p-values for those terms with corrected p-values less than 0.05.

Response: We have presented the KEGG analysis as a bar graph (Figure 6) in the revised manuscript.

13. A figure (like Fig. 6) showing the data on the HSP genes in the main text is recommended.

Response: We have changed the table to a figure showing the data on the HSP genes in the revised manuscript.

14. The RNA-seq data should be presented in a bar graph similarly to the qRT-PCR. Log transformation of the data is recommended.

Response: The RNA-seq data has been changed to a bar graph similarly to the qRT-PCR in the revised manuscript.

15. Why were the HSPs, TF and Ub-ligases of such interest to the authors. How may their expression contribute to heat stress resistance? What is the molecular function of these different classes of genes and how does this function contribute to resistance to heat stress? Are the heat stress responses in HR and HS jujube plants similar to other plants? What was the most important biological process or pathway identified in the study as associated with heat susceptibility or tolerance? What is the most important difference found between heat susceptible and heat resistant cultivars?

Response: According to the previous research results, analysis of the transcriptome profile in plant after heat treatment indicated that the HSP family and transcription factors (TFs) play a central role in responding to heat stress. Moreover, researchers have reported that Ub-ligases genes confers enhanced heat tolerance, so we’re interested in these genes, and we have also added relevant content in the discussion section in the revised manuscript.

**Reviewer #2: In this manuscript the authors compare gene expression analysis between two jujube cultivars under heat stress. The results of gene differential expression are coupled to a few additional observations for confirming the occurrence of heat stress during treatments at 45°C.**

1. How did the authors select ‘HR’ and ‘HS’ Ziziphus jujube cultivars?

Response: Ziziphus jujuba cv.‘HR’ and Ziziphus jujuba cv.‘HS’ were grown in the Forestry Management Station of Turpan city, Forestry and Grassland Administration of Turpan city, located in Turpan, Xinjiang province, China. ‘HR’ has been proven to be resistant to heat stress while ‘HS’ is susceptible.

2. How did they set the heat treatment? Why did they choose 45 °C for heat treatments?

Response: In recent years, with the global climate warming, extreme high temperature weather frequently occurs in Xinjiang, China, and many areas have experienced continuous high temperature weather exceeding 40 ℃, which has greatly affected the growth and yield of jujube trees. In the early stage, we have conducted an evaluation test on the heat resistance of jujube leaves, and determined the treatment temperature (45 °C).

3. How many plants did they heat-stress and from how many plants did they collect the material? In the results section they wrote that they performed three biological replicate, however it is not clear what they mean with biological replicate. They also wrote that the validation of DEGs was done in the same plant samples, but again it is not clear on how many plants.

Response: There were a total of 180 plants for ‘HR’ and ‘HS’ seedlings, respectively. Each treatment included three biological replications for each cultivar, and each biological replicate contained 15 plants. Plant samples were collected after the treatments started.

4. It is also unclear on how many plants they did the TEM observations.

Response: For ultra-structural observation, the sixth leaf from the top was removed from 4 random plants in each treatment time point (n=3) for each cultivar.

5. In results the authors report" the nucleoli of two jujube cultivars gradually disappeared as the heat

stress time extended, but the nuclear membranes remained intact...." What does this mean biologically. In this chapter the discussion is missing and it is scarse also in the two chapters describing transcription factors and HSP70. In my opinion a discussion on the functional outputs of DEGs must be added in the manuscript that tends to be too superficial.

Response: We have revised the discussion section in the revised manuscript, including physiochemical changes, transcription factors, HSP70 and ubiquitin-protein ligase genes.
