# Supplementary material for: Comparative transcriptome analysis uncovers different heat stress responses in heat-resistant and heat-sensitive jujube cultivars
Source: PLoS One. 2020 Sep 21;15(9):e0235763. doi: 10.1371/journal.pone.0235763 (PMC7505471; doi:10.1371/journal.pone.0235763)
Supplement: Attachment — Submitted filename: Response to Reviewers.docx [file pone.0235763.s010.docx]

Dear editors,

The authors are very thankful to you and the reviewers for the valuable comments and remarks regarding this manuscript. We have tried to address all comments and suggestions adequately. The requested alterations/corrections have been inserted directly into the manuscript and are also described below.

We are looking forward to having the opportunity to publish our work on your journal. Thank you for your help again!

Best regards!

**Comments from Reviewers:**

**Reviewer #1: The manuscript is much improved particularly the Discussion section. There are some further revisions recommended for this version.**

1. Abstract: A sentence on how stress was quantified in the study should be added. The section starting with “in total, 6887… 4064 genes were significantly different in ‘HR’ and ‘HS’, respectively.” should be replaced with sentences indicating the functions of the differentially expressed genes at each time point that were discovered in the GO and KEGG analyses.

Response: “In total, 6887 and 5077 differentially expressed genes were identified in ‘HR’ and ‘HS’, respectively. After 1d of heat stress, 1880 and 2390 genes presented significantly different expression in ‘HR’ and ‘HS’ compared with the control; after 3d of heat stress, 4350 and 2998 genes were differentially expressed in ‘HR’ and ‘HS’; after 5d of heat stress, expression of 5435 and 4064 genes were significantly different in ‘HR’ and ‘HS’, respectively. The expression pattern of nine genes was validated by qRT-PCR. After gene ontology and KEGG enrichment analysis, the DEGs encoding heat shock proteins, transcriptional factors, and ubiquitin-protein ligase genes were found to be closely involved in heat stress response” was changed to “A total of 6887 and 5077 differentially expressed genes were identified in ‘HR’ and ‘HS’ after 1d, 3d, and 5d of heat stress compared with the control treatment, GO and KEGG enrichment analysis revealed that some of the genes were highly enriched in oxidation-reduction process, response to stress, response to water deprivation, response to heat, carbon metabolism, protein processing in endoplasmic reticulum, and plant hormone signal transduction and may play vital roles in the heat stress response in jujube plants. Differentially expressed genes were identified in the two cultivars, including heat shock proteins, transcriptional factors, and ubiquitin-protein ligase genes. And the expression pattern of nine genes was also validated by qRT-PCR”.

2. The text is difficult to read in Figs 2-6.

Response: We have changed the text font on Figs 2-6 in the revised manuscript.

3. Figure 5, 6 and 9 Captions: Provide a description of what the axes are on these graphs.

Response: We have revised the axes on Figure 5, 6 and 9 in the revised manuscript.

**Figure 5. Scatterplot of enriched GO pathways between the two cultivars after heat stress.** (A), (C) and (E) represent enriched GO pathways of the DEGs in HR 1d vs. HR0d, HR 3d vs. HR0d, and HR 5d vs. HR0d, respectively; (B), (D) and (E) represent enriched GO pathways of the DEGs in HS 1d vs. HS0d, HS 3d vs. HS0d, and HS 5d vs. HS0d, respectively. The horizontal axes represent the enriched GO pathways, vertical axes represent the GeneRatio of each GO pathway. GeneRatio refers to the ratio of the number of DEGs enriched in certain GO pathway to the total number of differentially expressed genes. The greater the value is, the higher the number of DEGs is. The size of the dots indicates the number of DEGs enriched in certain pathway, and the color of the dots corresponds to the range of the q value (adjusted p value). Only the top 20 terms are listed here.

**Figure 6. Scatterplot of enriched KEGG pathways between the two cultivars after heat stress.** (A), (C) and (E) represent enriched KEGG pathways of the DEGs in HR 1d vs. HR0d, HR 3d vs. HR0d, and HR 5d vs. HR0d, respectively; (B), (D) and (E) represent enriched KEGG pathways of the DEGs in HS 1d vs. HS0d, HS 3d vs. HS0d, and HS 5d vs. HS0d, respectively. The horizontal axes represent the enriched KEGG pathways, vertical axes represent the number of DEGs enriched in each KEGG pathway. The bar indicates the number of DEGs enriched in certain pathway, and the color of the bars corresponds to the range of the q value (adjusted p value). The longer the bar is, the more the number of DEGs is. Only the top 20 terms are listed here.

**Figure 9. Expression analysis of nine selected DEGs in ‘HR’ and ‘HS’ after 0, 1, 3, 5d of heat stress.** (A) qRT-PCR results of nine DEGs. (B) mRNA expression levels of the same DEGs determined by RNA-seq, Log2(FPKM) was used to calculate the expression levels of genes. Horizontal axes represent 0, 1, 3, and 5 d (from left to right) of heat stress in HR and HS, the vertical axes represent the expression levels. The white bar presents the expression levels in ‘HR’, and the grey bar presents the expression levels in ‘HS’. The error bars represent SD (n=3).

4. Indicate what the columns of the supplementary files are in the caption included at the top of the excel spreadsheets.

Response: We have renamed the columns of the supplementary files clearly and added the captions at the top of the excel spreadsheets in the revised manuscript.

5.Some additional discussion of GO and KEGG analyses is recommended. For example, photosynthetic complex genes including the light harvesting complex are differentially expressed in HR, but HS in both GO and KEGG analysis.

Response: We have added some discussion of GO and KEGG analyses in the revised manuscript.

6. Line spacing is uneven in the document.

Response: We have changed the line spacing to the double-space paragraph format in the revised manuscript.

**Reviewer #2: The authors did not respond to my previous observation no. 5. In results the authors report" the nucleoli of two jujube cultivars gradually disappeared as the heat stress time extended, but the nuclear membranes remained intact". What does this mean biologically?**

**I think the authors should discuss this point and consider the literature available on nucleoli and stress.**

Response: We have discussed this point in the revised manuscript. The nucleolus has been considered as a sensor of cell stress since it can suffer alterations of morphology, area, and number per nucleus in response to stress (Boulon et al. 2010; Jiang et al. 2014; Stepinski. 2014). In our study, we found that the nucleoli of two jujube cultivars were sensitive to heat stress and gradually disappeared, which had been reported in cowpea cells after exposure to elevated temperature of 45 ℃ (Dylewski et al., 1991).

.
